# An Integrative Analysis of O6-Methylguanine-DNA Methyltransferase (MGMT) Methylation and the Single-Nucleotide Polymorphism (SNP) rs1625649 Reveals Distinct Survival Patterns in Glioblastoma: A Retrospective Study

**DOI:** 10.7759/cureus.92674

**Published:** 2025-09-18

**Authors:** Krachi Agarwal, Shivanjali Raghuvanshi, Shalini Bhalla, Snehkiran Raghuvanshi, Alok Singh, Ajay Singh, BK Ojha

**Affiliations:** 1 Pathology, King George's Medical University, Lucknow, IND; 2 Prosthodontics and Crown and Bridge, King George's Medical University, Lucknow, IND; 3 Neurosurgery, King George's Medical University, Lucknow, IND

**Keywords:** glioblastoma, mgmt promoter methylation, prognostic biomarker, snp rs1625649, survival analysis, temozolomide resistance

## Abstract

Background

O6-methylguanine-DNA methyltransferase (MGMT) promoter methylation is an established predictive and prognostic biomarker in glioblastoma (GBM). However, the influence of MGMT promoter single-nucleotide polymorphisms (SNPs), particularly rs1625649, on gene expression and patient outcomes remains unclear. This study evaluated the prognostic impact of MGMT methylation and SNP rs1625649 in GBM patients.

Methods

This retrospective analytical study included 66 histologically confirmed GBM patients (IDH1 R132H-negative), treated at a tertiary care center from 2018 to 2022. MGMT methylation was assessed using methylation-specific PCR, and SNP rs1625649 genotyping was performed by PCR-restriction fragment length polymorphism (PCR-RFLP). Survival analyses were conducted using Kaplan-Meier and Cox proportional hazards models. Key clinical variables, including age, Karnofsky performance status (KPS), extent of resection, and treatment data, were analyzed.

Results

MGMT promoter methylation was present in 56.1% of cases and was significantly associated with longer overall survival (OS: 86.4 days (≈2.9 months) vs. 35.0 days (≈1.2 months); p < 0.001). The SNP rs1625649 AA genotype was observed in 15.2% of patients and was associated with prolonged OS (130.8 days) compared to CA (64.2 days) and CC (47.0 days) (p = 0.002). Among methylated cases, the AA genotype conferred superior survival (160.7 days; p = 0.009). Multivariate Cox analysis confirmed MGMT methylation (HR = 0.46; p = 0.005) and the rs1625649 AA genotype (HR = 0.41; p = 0.040) as independent prognostic factors.

Conclusion

MGMT promoter methylation and the SNP rs1625649 AA genotype are independently associated with improved survival in GBM. These findings highlight the potential prognostic utility of incorporating MGMT SNP genotyping alongside methylation status in clinical practice. Further large-scale studies are warranted to validate these results.

## Introduction

Gliomas are the most frequent, heterogeneous group of primary intracranial neoplasms, representing nearly 80% of all malignant brain tumors [1]. They originate from glial progenitor or stem cells, such as astrocytes, oligodendrocytes, and ependymal cells. The 2016 World Health Organization (WHO) classification system stratified gliomas into Grades II (low-grade astrocytoma), III (anaplastic astrocytoma), and IV (glioblastoma, or GBM) [2]. However, the latest WHO Classification of Tumors of the Central Nervous System (CNS5, 2021) represents a paradigm shift by integrating molecular diagnostics with histopathological evaluation, thereby enhancing the accuracy of CNS tumor classification. According to WHO CNS5, adult-type diffuse gliomas are now classified primarily based on IDH mutation status, with IDH-wildtype GBMs being the most aggressive form (CNS WHO Grade 4). Notably, the presence of any one of three genetic alterations - TERT promoter mutation, EGFR amplification, or combined gain of chromosome 7 and loss of chromosome 10 (+7/-10) - is sufficient for diagnosing GBM in IDH-wildtype diffuse astrocytic tumors, even in the absence of histological features such as necrosis or microvascular proliferation [3].

Despite aggressive multimodal therapy, GBM remains incurable, with a median survival of approximately 15 months. Surgical resection, followed by concurrent chemoradiotherapy using temozolomide (TMZ), known as the Stupp regimen, is the current standard of care [4]. TMZ exerts its cytotoxic effect by adding methyl groups to DNA bases, particularly at the O6 position of guanine. This leads to DNA mismatch repair failure and apoptotic cell death [5]. However, this effect is counteracted by O6-methylguanine-DNA methyltransferase (MGMT), a DNA repair protein encoded by the MGMT gene on chromosome 10q. MGMT promoter methylation silences gene expression, thereby enhancing sensitivity to TMZ and improving survival outcomes [6]. Consequently, MGMT methylation status serves as both a predictive and prognostic biomarker in GBM [7]. However, genetic polymorphisms in the MGMT promoter region may also influence gene expression and therapeutic response [8]. Among these, the rs1625649 single-nucleotide polymorphism (SNP) has been reported as functionally relevant, with studies showing its role in downregulating MGMT protein expression, particularly in MGMT-methylated tumors [9]. This SNP has also been investigated in other malignancies, such as lung adenocarcinoma and colorectal cancer [10].

Emerging evidence suggests that GBM patients with concurrent MGMT methylation and SNP rs1625649 AA genotype may derive the greatest benefit from TMZ therapy, demonstrating significantly longer progression-free survival (PFS) and overall survival (OS) [11]. Furthermore, the interplay between MGMT methylation, SNPs, and transcription factors such as NF-κB and SP1 has been shown to modulate MGMT expression at the promoter level [12]. While global studies have explored MGMT methylation and SNP variants, there is a paucity of data in the Indian population, particularly concerning SNP rs1625649. Considering population-specific genetic variability and its potential clinical relevance, our study aims to address this critical gap.

The present study was designed to determine the frequency of MGMT promoter methylation and rs1625649 SNP genotypes (AA, CA, and CC) in histologically confirmed GBM patients, and to evaluate their association with clinical outcomes, specifically PFS and OS. In addition, the study aimed to assess the prognostic impact of rs1625649 genotypes on survival and to investigate the combined effect of MGMT promoter methylation and SNP status in predicting outcomes. Finally, the study sought to explore the clinical utility of integrating SNP genotyping with MGMT methylation testing as a dual-biomarker approach for prognostication in the Indian GBM patient population.

## Materials and methods

Study design and setting

This retrospective, laboratory-based analytical study was conducted in the Department of Pathology, in collaboration with the Departments of Neurosurgery and Radiotherapy at King George’s Medical University (KGMU), Lucknow, India. The study aimed to evaluate the prognostic relevance of MGMT promoter methylation and promoter SNP rs1625649 in GBM patients. Ethical approval was obtained from the Institutional Ethics Committee (approval no. 536/ethics/2021; dated June 2, 2021).

Study population and sample size

Sixty-six histopathologically confirmed GBM (WHO Grade 4) cases diagnosed between January 2021 and July 2023 were included. The sample size was calculated using Cox proportional hazards survival analysis, assuming α = 0.05, β = 0.20 (80% power), equal group proportions, and a hazard ratio of 0.49 based on prior literature [13-15]. The required sample size was 62, and 66 patients were included, exceeding this minimum.

Inclusion/exclusion criteria

Eligible patients were those with histopathologically confirmed GBM and adequate tissue for molecular analysis. Patients with coexistent malignancies, or with inadequate tissue material, were excluded. Clinical, radiological, and treatment details were retrieved from medical records.

Histopathological and molecular classification

Diagnosis was confirmed on H&E-stained sections, with characteristic features including palisading necrosis and/or microvascular proliferation. Immunohistochemistry for the IDH1 R132H mutation was performed (anti-IDH1 R132H clone H09, Dianova); all cases were IDH1-negative, confirming IDH-wildtype GBM. Due to retrospective constraints, additional markers required under WHO CNS5 (e.g., EGFR amplification, +7/-10 signature, TERT mutations) could not be assessed.

DNA extraction and quantification

Genomic DNA was extracted from FFPE tumor tissues using the Qiagen FFPE DNA Kit (Qiagen, Germantown, MD, USA), after macrodissection to enrich tumor regions (>10% viable cells). DNA concentration was measured with the Qubit® 4.0 Fluorometer (Life Technologies, Carlsbad, CA, USA). For SNP genotyping, DNA from peripheral blood leukocytes was extracted using phenol-chloroform or Qiagen kits. DNA quality was verified (A260/A280 = 1.8-2.0).

Bisulfite conversion and methylation-specific PCR (MSP)

For methylation analysis, 500 ng of DNA were bisulfite-converted using Zymo Research kits (Zymo Research Corporation, Irvine, CA, USA). MSP was performed in 20 µL reactions, with methylation- and unmethylation-specific primers. Amplification conditions included 40 cycles (95°C for 30 seconds, 65°C for 30 seconds, and 72°C for 30 seconds). Products were resolved on 2.5% agarose gels and visualized using the Bio-Rad Gel Doc system (Bio‑Rad Laboratories, Inc., Hercules, CA, USA). Methylation was indicated by 81 bp bands (MGMT-M primers), and unmethylation by 93 bp bands (MGMT-U primers).

Genotyping of the rs1625649 SNP

Primers flanking rs1625649 were designed (forward: 5′-TGTCCCTTCACCAGAGTGAC-3′; reverse: 5′-GCTGCTCCAGGAAAGGAAAC-3′), generating a 173 bp fragment. PCR reactions (25 µL) included 12.5 µL of 2× PCR Master Mix (Thermo Scientific, Waltham, MA, USA), primers (0.8 µL each, 20 pmol/µL), and 5 µL of DNA. Conditions: 95°C for two minutes; 35 cycles of 95°C for 30 seconds, 56°C for 40 seconds, and 72°C for 30 seconds; final extension at 72°C for five minutes. Products were digested with 5 U BsaJI at 55°C for four hours and resolved on 3% agarose gels. Genotype assignment: CC (173 bp), AA (151 bp + faint 22 bp), CA (both 173 bp and 151 bp).

Assay validation and controls

Methylated and unmethylated DNA standards were used as positive and negative controls in MSP. For SNP genotyping, 10% of cases were randomly re-genotyped, with 100% concordance observed. No Sanger sequencing confirmation was performed, which is acknowledged as a limitation.

Clinical data and follow-up

Demographic data, presenting symptoms, imaging findings, extent of surgical resection, adjuvant therapy (radiotherapy and TMZ cycles), and survival status were retrieved from medical records and follow-up visits. The extent of resection was classified as gross total resection (GTR), subtotal resection, or biopsy only. Radiotherapy was administered as 60 Gy in 30 fractions over six weeks, where feasible. TMZ was prescribed at 150-200 mg/m² for five days in 28-day cycles. Only 14 patients (21.2%) completed at least one full TMZ cycle. Follow-up duration ranged from 2 to 24 months, with a median of 8.5 months. OS was defined as the time from surgery to death or last follow-up.

Statistical analysis

All statistical analyses were conducted using IBM SPSS Statistics for Windows, Version 26 (Released 2019; IBM Corp., Armonk, NY, USA). Descriptive statistics were used to summarize patient characteristics, including frequencies and percentages for categorical variables, and means with standard deviations or medians with ranges for continuous variables. Comparative analysis of clinical and pathological characteristics across MGMT methylation status (methylated vs. unmethylated) and SNP rs1625649 genotypes (AA, CA, and CC) was performed using the Chi-square test for categorical variables, and ANOVA or Kruskal-Wallis tests for continuous variables, as appropriate. A p-value <0.05 was considered statistically significant. Survival analysis was performed using the Kaplan-Meier method, and differences in survival distributions were compared using the log-rank (Mantel-Cox) test. OS was defined as the time from surgical resection to death from any cause or last follow-up. The results were presented as mean OS with 95% confidence intervals (CI). Subgroup analyses were conducted to evaluate OS based on MGMT methylation status, SNP rs1625649 genotype, and their combined effects, as well as in TMZ-treated patients. To evaluate the independent prognostic value of MGMT methylation and SNP rs1625649, a Cox proportional hazards regression model was constructed. The model included the following covariates: age group (≤40 vs. >40 years), Karnofsky performance status (KPS) (≥70 vs. <70), extent of resection (gross total vs. subtotal/biopsy), radiotherapy (yes/no), number of TMZ cycles (as a continuous variable), and MGMT methylation status.

## Results

Patient demographics and clinicopathological characteristics

The demographics and clinicopathological characteristics are depicted in Table 1. The study cohort included 66 patients diagnosed with GBM. The median age was >40 years, with 60.6% (n = 40) of patients above 40 years and 39.4% (n = 26) aged 40 years or below. Males predominated the study population (62.1%, n = 41), with females comprising 37.9% (n = 25). Common presenting symptoms included focal neurological deficits in 66.7% (n = 44), headache in 53.0% (n = 35), and seizures in 39.4% (n = 26), while loss of consciousness was noted in 24.2% (n = 16). Radiologically, the majority had supratentorial GBMs (97.0%), with lesions most frequently involving the parietal/temporal lobes (36.4%), followed by other lobes or thalamus (43.9%), and the frontal lobe (19.7%). A midline shift was observed in 87.9% (n = 58) of patients, with a mean shift of 8.46 ± 2.66 mm. The KPS was ≥70 in 51.5% (n = 34) and <70 in 48.5% (n = 32). In terms of surgical management, GTR was achieved in 36.4% (n = 24), subtotal resection in 53.0% (n = 35), and biopsy only in 10.6% (n = 7). Adjuvant radiotherapy was administered to 66.7% (n = 44) of patients, with a median dose of 60 Gy, delivered in 2.0 Gy fractions over six weeks (5 fractions/week). TMZ chemotherapy was given to 21.2% (n = 14), with a median of three cycles (range 1-6). Molecular analysis revealed MGMT promoter methylation in 56.1% (n = 37) and unmethylated status in 43.9% (n = 29). Genotyping for SNP rs1625649 showed AA genotype in 15.2%, CA in 22.7%, and CC in 62.1% of patients. IDH1 (R132H) mutation testing by immunohistochemistry was negative in all cases (100%), and EGFR amplification and +7/-10 chromosomal signature were not assessed. The median follow-up duration was 8.5 months (range: 2-24 months). At the last follow-up, 93.9% (n = 62) of the patients had died, while only 6.1% (n = 4) were alive.

**Table 1:** Clinicopathological Characteristics of the Study Population

Characteristic	Category	No. (%)
Age group (years)	≤ 40	26 (39.4%)
	> 40	40 (60.6%)
Gender	Male	41 (62.1%)
	Female	25 (37.9%)
Presenting symptoms	Headache	35 (53.0%)
	Seizures	26 (39.4%)
	Focal neurological deficit	44 (66.7%)
	Loss of consciousness	16 (24.2%)
Radiological features	Supratentorial glioblastoma	64 (97.0%)
	Frontal lobe	13 (19.7%)
	Parietal/temporal lobes	24 (36.4%)
	Other lobes/thalamus	29 (43.9%)
	Midline shift present	58 (87.9%)
	Mean midline shift (mm)	8.46 ± 2.66
Karnofsky performance score (KPS)	≥ 70	34 (51.5%)
	< 70	32 (48.5%)
Extent of resection	Gross total resection (GTR)	24 (36.4%)
	Subtotal resection	35 (53.0%)
	Biopsy only	7 (10.6%)
Radiotherapy received	Yes	44 (66.7%)
	No	22 (33.3%)
Radiotherapy dose	Median dose	60 Gy (2.0 Gy/fraction, 5 fractions/week for 6 weeks)
Temozolomide received	Yes	14 (21.2%)
	No	52 (78.8%)
Temozolomide cycles	Median number of cycles (range)	3 (1-6)
O6-methylguanine-DNA methyltransferase (MGMT) methylation status	Methylated	37 (56.1%)
	Unmethylated	29 (43.9%)
Single-nucleotide polymorphism (SNP) rs1625649 genotype	AA	10 (15.2%)
	CA	15 (22.7%)
	CC	41 (62.1%)
IDH1 (R132H) status (IHC)	Negative	66 (100%)
Follow-up duration	Median (range)	8.5 months (2-24)
Overall survival status	Alive	4 (6.1%)
	Died	62 (93.9%)

Association of clinical parameters with MGMT methylation status and SNP rs1625649 genotype

The association of clinical and treatment-related variables with MGMT promoter methylation status and SNP rs1625649 genotypes was analyzed and is summarized in Table 2. No statistically significant associations were found between MGMT methylation and age group (p = 0.460 for ≤40 years; p = 0.442 for >40 years), gender (p = 0.314 for males; p = 0.128 for females), or presenting symptoms such as headache (p = 0.675), seizures (p = 0.736), focal neurological deficit (p = 0.310), or loss of consciousness (p = 0.870). Similarly, functional status, as assessed by KPS ≥70 vs. <70, was not significantly different between methylated and unmethylated groups (p = 0.790 and p = 0.958, respectively). Surgical parameters such as extent of resection (GTR: p = 0.911; subtotal resection: p = 1.000; biopsy only: p = 1.000), receipt of radiotherapy (p = 0.310), and administration of TMZ (p = 0.672) also did not show significant associations with MGMT methylation. Similarly, no statistically significant associations were observed between SNP rs1625649 genotypes (AA, CA, and CC) and clinical features. The p-values for comparisons with age (p = 0.908 for ≤40 years; p = 0.938 for >40 years), gender (p = 0.985 for males; p = 0.975 for females), headache (p = 0.895), seizures (p = 0.825), focal neurological deficit (p = 0.931), and loss of consciousness (p = 0.945) were all non-significant. KPS distribution was also not different among the genotypes (p = 0.992 for both KPS ≥70 and <70). No significant differences were observed across SNP genotypes with respect to the extent of resection (gross total: p = 0.927; subtotal: p = 0.898; biopsy: p = 0.933), radiotherapy (p = 0.957), or TMZ administration (p = 0.113 for received; p = 0.535 for not received). Overall, neither MGMT methylation status nor SNP rs1625649 genotype showed any statistically significant association with demographic, clinical, radiological, or treatment variables, supporting their potential role as independent molecular predictors in GBM.

**Table 2:** Association of Clinical Parameters With MGMT Methylation Status and SNP rs1625649 Genotype (n = 66) MGMT, O6-methylguanine-DNA methyltransferase; SNP, single-nucleotide polymorphism; LOC, loss of consciousness; KPS, Karnofsky performance status; GTR, gross total resection; STR, subtotal resection; Bx, biopsy

Parameter	MGMT Methylated	MGMT Unmethylated	Test Used	Test Statistic	p-value (MGMT)	AA	CA	CC	Test Used	Test Statistic	p-value (SNP)
Age ≤40/>40	12/25	14/26	χ²	χ² = 0.55	0.460	4/6	5/10	17/24	χ²	χ² = 0.20	0.908
Gender (M/F)	19/18	22/7	χ²	χ² = 1.02	0.314	6/4	9/6	26/15	χ²	χ² = 0.01	0.985
Headache (Y/N)	21/16	14/15	χ²	χ² = 0.18	0.675	6/4	7/8	22/19	χ²	χ² = 0.02	0.895
Seizures (Y/N)	13/24	13/16	χ²	χ² = 0.11	0.736	5/5	6/9	15/26	χ²	χ² = 0.05	0.825
Focal deficit (Y/N)	28/9	16/13	χ²	χ² = 1.03	0.310	7/3	9/6	28/13	χ²	χ² = 0.01	0.931
LOC (Y/N)	8/29	8/21	Fisher	OR = 0.96	0.870	2/8	4/11	10/31	Fisher	OR = 0.98	0.945
KPS ≥70/<70	20/17	14/15	χ²	χ² = 0.07	0.790	5/5	8/7	21/20	χ²	χ² = 0.00	0.992
Resection (GTR/STR/Bx)	14/19/4	10/16/3	Fisher	-	0.911	4/5/1	6/7/2	14/23/4	Fisher	-	0.927
Radiotherapy (Y/N)	28/9	16/13	χ²	χ² = 1.03	0.310	6/4	10/5	28/13	χ²	χ² = 0.00	0.957
Temozolomide (Y/N)	9/28	5/24	Fisher	-	0.672	4/6	5/10	5/36	Fisher	-	0.113

Survival analysis based on MGMT methylation and SNP rs1625649 genotype

**Figure 1:**
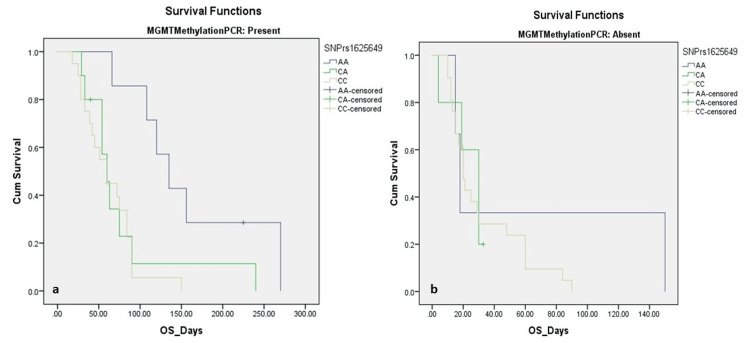
Kaplan-Meier Survival Analysis for Overall Survival in Glioblastoma Patients (n = 66) (a) OS, stratified by MGMT promoter methylation status. Patients with a methylated MGMT promoter had significantly longer mean OS than unmethylated cases (86.38 vs. 35.03 days; p < 0.001). (b) OS, stratified by rs1625649 genotypes. Patients with the AA genotype had the longest OS (130.8 days), followed by CA (64.2 days) and CC (46.9 days) (p = 0.002). MGMT, O6-methylguanine-DNA methyltransferase; OS, overall survival

Kaplan-Meier survival analysis revealed a significant association between MGMT promoter methylation status and OS, as depicted in Table 3. Patients with MGMT methylation had a significantly longer mean OS of 86.38 ± 10.77 days (95% CI: 65.26-107.49), compared to those with unmethylated MGMT, who had a mean OS of 35.03 ± 6.26 days (95% CI: 22.77-47.29) (log-rank χ² = 19.183, p < 0.001) (Figure 1a). When stratified by SNP rs1625649 genotype, the mean OS varied significantly across groups (χ² = 12.098, p = 0.002) (Figure 1b). Patients with the AA genotype showed the longest survival (130.80 ± 28.21 days; 95% CI: 75.51-186.09), followed by the CA genotype (64.18 ± 17.15 days; 95% CI: 30.55-97.81), and the CC genotype (46.97 ± 5.11 days; 95% CI: 36.96-56.99).

**Table 3:** Association of Overall Survival With MGMT Methylation Status and SNP rs1625649 Genotype (Kaplan-Meier Analysis, n = 66) MGMT, O6-methylguanine-DNA methyltransferase; SNP, single-nucleotide polymorphism; TMZ, temozolomide

Group	Subgroup	n	Deaths	Mortality %	Mean OS (days)	SE	95% CI (Lower - Upper)	Log-Rank χ²	p-value
MGMT methylation status	Methylated	37	34	91.9%	86.38	10.77	65.26 - 107.49	19.183	<0.001
	Unmethylated	29	28	96.6%	35.03	6.26	22.77 - 47.29		
SNP rs1625649 (all cases)	AA	10	9	90.0%	130.80	28.21	75.51 - 186.09	12.098	0.002
	CA	15	13	86.7%	64.18	17.15	30.55 - 97.81		
	CC	41	40	97.6%	46.97	5.11	36.96 - 56.99		
SNP rs1625649 among MGMT methylated	AA (n = 7)	7	6	85.7%	160.71	30.50	100.94 - 220.49	9.535	0.009
	CA (n = 10)	10	9	90.0%	78.89	21.46	36.83 - 120.94		
	CC (n = 20)	20	19	95.0%	62.79	7.47	48.16 - 77.43		
SNP rs1625649 among MGMT unmethylated	AA (n = 3)	3	3	100.0%	61.00	44.51	0.00 - 148.24	0.907	0.635
	CA (n = 5)	5	4	80.0%	23.20	4.79	13.80 - 32.60		
	CC (n = 21)	21	21	100.0%	31.95	5.39	21.38 - 42.53		
MGMT methylation in TMZ-treated patients	Methylated	9	6	66.7%	139.13	36.31	67.96 - 210.29	1.158	0.282
	Unmethylated	5	4	80.0%	82.40	26.99	29.49 - 135.31		

**Figure 2:**
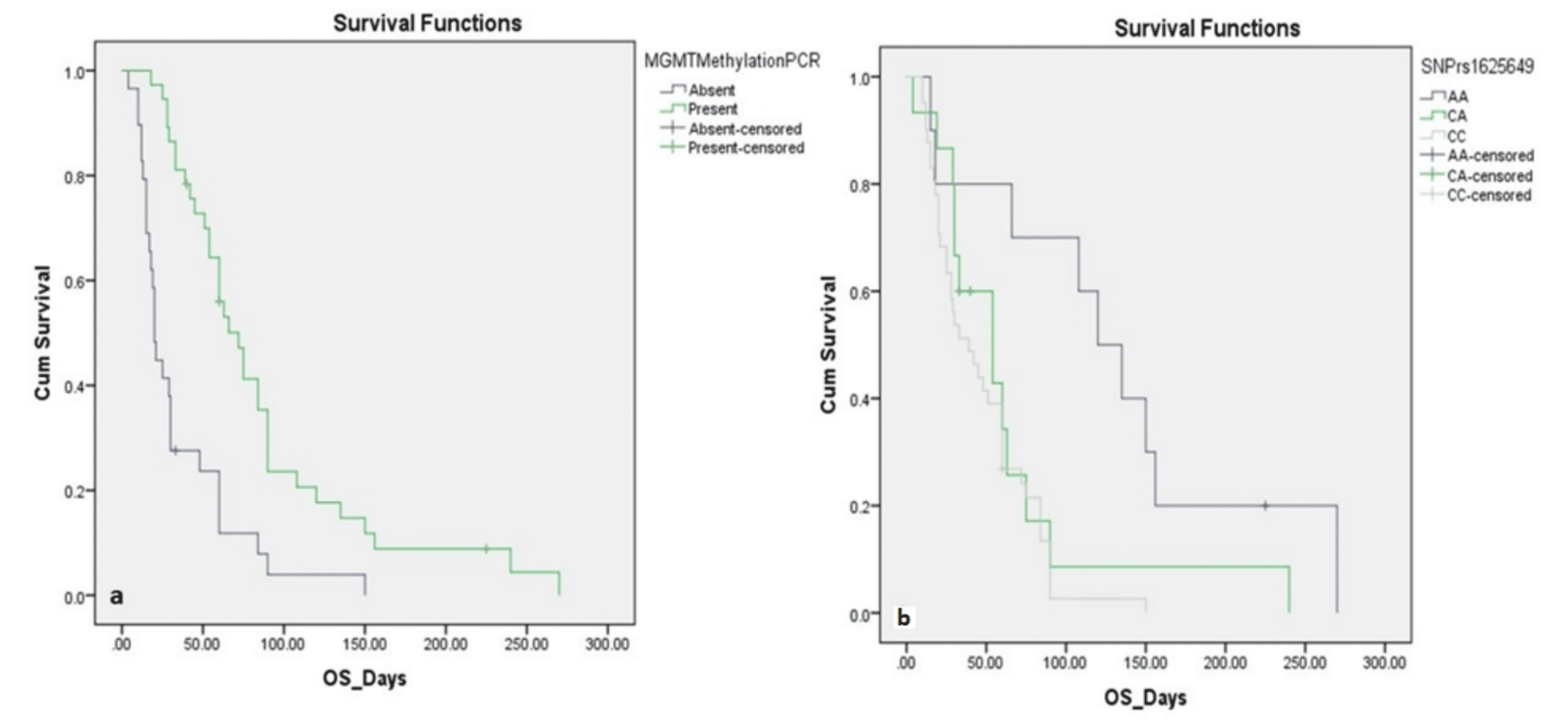
Kaplan-Meier Survival Analysis for Overall Survival in Glioblastoma Patients (n = 66) (a) OS among MGMT-methylated cases, by rs1625649 genotype. The AA genotype showed the longest PFS (160.7 days), compared to CA (78.9 days) and CC (62.8 days; p = 0.009). (b) OS among MGMT-unmethylated cases, by rs1625649 genotype. No significant differences were observed (p = 0.635). MGMT, O6-methylguanine-DNA methyltransferase; OS, overall survival; PFS, progression-free survival

**Figure 3:**
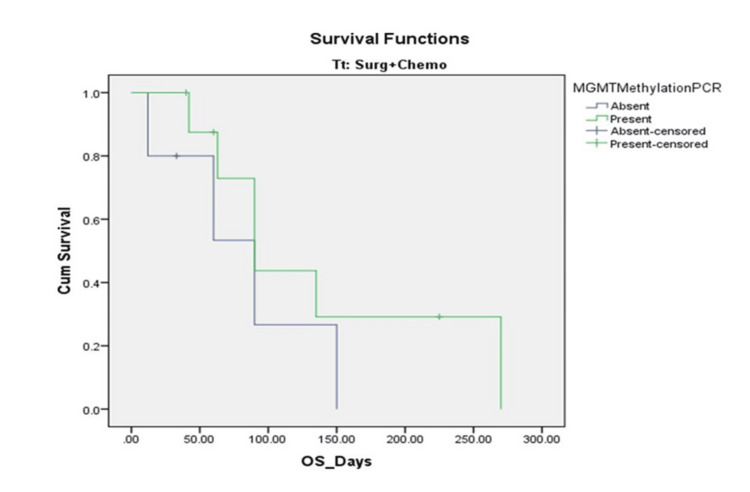
Kaplan-Meier Survival Analysis for Overall Survival in Glioblastoma Patients (n = 66) OS in temozolomide-treated patients, stratified by MGMT methylation status. Methylated patients had a higher mean OS (139.13 vs. 82.40 days), although this was not statistically significant (p = 0.282). MGMT, O6-methylguanine-DNA methyltransferase; OS, overall survival

In the subgroup of MGMT-methylated patients, the association between SNP rs1625649 genotype and survival remained statistically significant (χ² = 9.535, p = 0.009) (Figure 2a). Those with the AA genotype had a markedly prolonged mean OS of 160.71 ± 30.50 days (95% CI: 100.94-220.49), compared to 78.89 ± 21.46 days (95% CI: 36.83-120.94) in CA, and 62.79 ± 7.47 days (95% CI: 48.16-77.43) in CC genotype carriers. However, among the MGMT-unmethylated group, there was no significant difference in survival across SNP genotypes (χ² = 0.907, p = 0.635) (Figure 2b). The mean OS was 61.00 ± 44.51 days for AA (95% CI: 0.00-148.24), 23.20 ± 4.79 days for CA (95% CI: 13.80-32.60), and 31.95 ± 5.39 days for CC (95% CI: 21.38-42.53). In a subgroup of patients who received TMZ chemotherapy, MGMT methylation again appeared to influence survival, although this difference was not statistically significant. The mean OS for methylated cases was 139.13 ± 36.31 days (95% CI: 67.96-210.29), compared to 82.40 ± 26.99 days (95% CI: 29.49-135.31) in unmethylated cases (χ² = 1.158, p = 0.282) (Figure 3). These findings suggest that both MGMT promoter methylation and SNP rs1625649, particularly the AA genotype, may have prognostic relevance in GBM, especially among patients with methylated MGMT.

Multivariable survival analysis using the Cox proportional hazards model

To identify independent predictors of OS, a multivariate Cox proportional hazards regression analysis was performed, including clinically relevant covariates (Table 4). The analysis demonstrated that MGMT promoter methylation was independently associated with a significantly lower hazard of death (HR = 0.46, 95% CI: 0.27-0.79, p = 0.005), indicating a protective effect on survival. Among genotypic variants of SNP rs1625649, patients with the AA genotype had a significantly reduced risk of death compared to the CC genotype (HR = 0.41, 95% CI: 0.17-0.96, p = 0.040). The CA genotype also showed a trend toward lower hazard compared to CC, but this was not statistically significant (HR = 0.77, 95% CI: 0.39-1.52, p = 0.450). Other clinical variables, including age >40 years (HR = 1.28, p = 0.395), KPS <70 (HR = 1.62, p = 0.085), extent of resection (subtotal/biopsy vs. gross total) (HR = 1.45, p = 0.210), receipt of radiotherapy (HR = 0.74, p = 0.302), and number of TMZ cycles (HR per cycle = 0.88, p = 0.239), did not show statistically significant associations with OS in this model. These findings support the role of MGMT methylation status and rs1625649 AA genotype as potential independent prognostic markers in GBM patients, after adjusting for key clinical and therapeutic variables.

**Table 4:** Cox Proportional Hazards Model for Overall Survival (n = 66) MGMT, O6-methylguanine-DNA methyltransferase; SNP, single-nucleotide polymorphism; KPS, Karnofsky performance status; GTR, gross total resection; TMZ, temozolomide

Variable	Hazard Ratio (HR)	95% CI	p-value
MGMT methylation (yes)	0.46	0.27 - 0.79	0.005
SNP rs1625649 (AA vs. CC)	0.41	0.17 - 0.96	0.040
SNP rs1625649 (CA vs. CC)	0.77	0.39 - 1.52	0.450
Age > 40 years	1.28	0.72 - 2.27	0.395
KPS < 70	1.62	0.94 - 2.78	0.085
Subtotal/biopsy vs. GTR	1.45	0.81 - 2.59	0.210
Radiotherapy (yes)	0.74	0.41 - 1.32	0.302
TMZ cycles (per cycle)	0.88	0.71 - 1.09	0.239

## Discussion

MGMT promoter methylation is a well-established predictive and prognostic biomarker in GBM, particularly in patients treated with alkylating agents like TMZ. Epigenetic silencing of MGMT reduces DNA repair capability, enhancing sensitivity to DNA-damaging agents. The prognostic role of SNPs within the MGMT promoter region, such as rs1625649, is a growing area of interest in neuro-oncology. In the present cohort of 66 histologically confirmed IDH1-negative GBM cases, MGMT promoter methylation was identified in 56.1% of patients, consistent with rates reported in earlier studies (~40%-60%) [16,17]. Female patients showed a higher proportion of MGMT methylation (48.6%) than males (32.8%), though the association did not reach statistical significance (p = 0.128). Similarly, no significant association was observed between MGMT methylation and other baseline clinical parameters, including age, KPS, extent of resection, and TMZ use.

Importantly, Kaplan-Meier analysis demonstrated significantly improved OS in patients with MGMT methylation compared to unmethylated cases (median OS: 86.38 vs. 35.03 days, p < 0.001). This finding aligns with previous prospective trials and meta-analyses, confirming the prognostic relevance of MGMT methylation, even in suboptimally treated cohorts [18,19]. We also examined the distribution and impact of the MGMT promoter SNP rs1625649, observing AA, CA, and CC genotypes in 15.2%, 22.7%, and 62.1% of patients, respectively. Although genotype frequencies did not differ significantly across clinical variables, survival outcomes varied notably. Patients with the AA genotype demonstrated significantly longer mean OS (130.8 days), compared to the CA (64.18 days) and CC (46.97 days) genotypes (p = 0.002). Among MGMT-methylated cases, the survival advantage conferred by the AA genotype remained significant (mean OS: 160.71 vs. 78.89 vs. 62.79 days for AA, CA, and CC; p = 0.009), but was not observed in the unmethylated subgroup (p = 0.635). These findings suggest a potential synergistic prognostic role of MGMT methylation and the rs1625649 AA genotype.

Multivariate Cox proportional hazards analysis further supported the independent prognostic significance of both MGMT methylation (HR = 0.46, p = 0.005) and rs1625649 AA genotype (HR = 0.41, p = 0.040), after adjusting for age, KPS, extent of resection, radiotherapy, and TMZ cycles. Notably, classical prognostic indicators such as age > 40 years, KPS < 70, and incomplete resection were not significantly associated with survival in the adjusted model, potentially due to the modest sample size and low treatment uniformity. The reported survival estimates in this study are shorter than expected from standard-of-care-treated GBM patients (Stupp regimen). This discrepancy reflects real-world limitations in our setting: only 21.2% of patients received TMZ, and radiotherapy was administered to 66.7% of the cohort. Financial constraints, non-compliance, and late presentation contributed to heterogeneous treatment and early mortality. Nevertheless, even in this resource-limited cohort, the statistically significant survival advantage of MGMT methylation and the rs1625649 AA genotype supports their robust prognostic utility, independent of therapy. These results are consistent with findings by Hsu et al. (2017), who reported improved PFS in patients with the rs1625649 AA genotype [8]. Conversely, in non-CNS malignancies such as colorectal and lung cancers, this SNP has shown variable or even inverse effects, indicating possible tumor-type specificity in its biological role [20,21].

While our findings support the prognostic utility of MGMT promoter methylation and the rs1625649 SNP, the question of clinical translation is equally important. SNP testing by PCR-based methods is relatively inexpensive compared to next-generation sequencing, and can be performed in most molecular pathology laboratories already equipped for MGMT methylation analysis. The additional cost per sample is modest, and turnaround time is short, which makes incorporation into routine diagnostic workflows feasible. In resource-limited settings, where comprehensive molecular profiling is often not available, targeted SNP genotyping could serve as a pragmatic surrogate biomarker to refine prognostic stratification. In the future, prospective validation in larger cohorts and cost-effectiveness studies will be needed to establish standardized protocols. Integration of rs1625649 testing with existing MGMT methylation assays could create a dual-marker panel, enhancing predictive accuracy for patient counseling and treatment planning without imposing prohibitive financial or logistical burdens.

The main limitations of this study include the relatively small sample size, particularly in subgroup analyses (e.g., n = 10 for the AA genotype), which reduced statistical power (post hoc power = 61.5%, below the conventional 80% threshold), and makes these findings exploratory. In addition, comprehensive molecular profiling, as recommended by the WHO 2021 classification of GBM, was not performed. Important markers such as EGFR amplification, TERT promoter mutations, and the combined whole chromosome +7/-10 signature were not assessed, which may have led to partial diagnostic stratification and limited comparability with fully characterized cohorts. Furthermore, MGMT copy number changes and protein expression were not evaluated, and the SNP genotyping protocol lacked undigested control lanes in gel images, reducing analytical reproducibility. Despite these limitations, the study underscores the potential value of combining MGMT promoter methylation with rs1625649 SNP analysis for prognostication in GBM, especially in low-resource settings where access to comprehensive molecular testing and full adjuvant therapy is limited. This dual biomarker approach could refine survival estimates and inform clinical decision-making in such contexts.

## Conclusions

This study demonstrates that MGMT promoter methylation and the rs1625649 AA genotype are associated with improved OS in GBM patients, with the strongest effect observed in the methylated subgroup. These findings suggest that SNP genotyping, when combined with MGMT methylation analysis, may enhance prognostic stratification. However, given the modest sample size, incomplete molecular profiling, and treatment heterogeneity, the results should be interpreted as preliminary. Larger, well-designed prospective studies are required to validate these associations and to determine whether integrating SNP testing into prognostic models provides clinical benefit in GBM management.
